# Up-regulation of *miR-187* modulates the advances of oral carcinoma by targeting *BARX2* tumor suppressor

**DOI:** 10.18632/oncotarget.11349

**Published:** 2016-08-17

**Authors:** Shu-Chun Lin, Shou-Yen Kao, Jennifer Chen-Yu Chang, Ying-Chieh Liu, En-Hao Yu, Ssu-Hsueh Tseng, Chung-Ji Liu, Kuo-Wei Chang

**Affiliations:** ^1^ Institute of Oral Biology, National Yang-Ming University, Taipei, Taiwan; ^2^ Department of Dentistry, School of Dentistry, National Yang-Ming University, Taipei, Taiwan; ^3^ Department of Stomatology, Taipei Veterans General Hospital, Taipei, Taiwan; ^4^ Department of Dentistry, MacKay Memorial Hospital, Taipei, Taiwan

**Keywords:** BARX2, carcinoma, invasion, metastasis, miR-187

## Abstract

Oral squamous cell carcinoma (OSCC) is one of the most common cancers worldwide. Aberrations in miRNA regulation are known to play important roles in OSCC pathogenesis. *miR-187* was shown to be up-regulated in head and neck malignancies in our previous screening. This study further investigated the oncogenic potential, clinical implications, and targets of *miR-187* in OSCC. We observed that *miR-187* increased oncogenicity, particularly migration, of OSCC cells. *miR-187* expression increased the xenografic tumorigenicity and metastasis in mice. In addition, metastatic human OSCC had higher *miR-187* expression than did non-metastatic tumors. Through vigorous screening, we confirmed BarH-like Homeobox 2 (*BARX2*) gene as an *miR-187* target. BARX2 expression suppressed the migration, invasion, anchorage-independent colony formation, and orthotopic tumorigenesis of OSCC cells. The migratory phenotype and neck metastasis induced by *miR-187* was rescued by BARX2 expression. BARX2 expression was down-regulated in the vast majority of OSCC, and this down-regulation was particularly conspicuous in tumors with advanced nodal metastasis. In addition, plasma *miR-187* was significantly higher in OSCC patients than in normal individuals. This study highlights the roles of *miR-187*-BARX2 in driving the carcinogenesis of OSCC. The results suggest that *miR-187* is a potential serological marker for OSCC and that targeting of *miR-187* might prove effective in attenuating nodal metastasis.

## INTRODUCTION

Head and neck squamous cell carcinoma (HNSCC), including oral SCC (OSCC), is the 6th most prevalent malignancy worldwide [1–3]. Previous studies have shown that microRNAs (miRNAs) can control the initiation or progression of HNSCC or OSCC, acting either as oncogenes or tumor suppressors [2–10]. MicroRNAs are a class of short, endogenous, non-coding RNAs that negatively regulate target gene expression [11, 12]. As of today, there are 1881 predicted miRNA species in human in miRBase database (version 21). One individual miRNA can regulate a diverse set of target genes, with approximately 60% of the human protein-coding genes regulated by miRNAs. Thus, miRNAs play crucial roles in the modulation of physiological processes and pathogenesis [13]. Disruption in miRNA expression could affects the malignancy and survival of OSCC by interfering with the regulation of processes such as apoptosis, invasion, drug resistance, stemness, hypoxia and signaling pathways, and others [4, 7, 14].

The *miR-187* gene is located on chromosome 18q12.2. Its overexpression has been observed in thyroid cancer, possibly serving as a clinical diagnostic marker [15], in breast cancer,, where it has been demonstrated and has been shows to is correlated with a more aggressive, invasive cancer phenotype as well as poor patient outcome and lower survival [16]. *miR-187* also represses the tumor-suppressor gene disabled homolog-2 (*Dab2*) in ovarian cancers [17]. Increased *miR-187* expression was observed in patients with ovarian and gall bladder cancer [18, 19]. In addition, low *miR-187* expression defines the sensitivity of ovarian cancers to taxol therapy [20]. However, studies have also revealed that *miR-187* is suppressive to some malignancies, including prostate and pancreatic carcinomas as well as clear renal cell carcinoma [21–25]. Therefore, the functional role of *miR-187* in different cancers may be paradoxical. In addition to its role in oncogenesis, *miR-187* takes part in the regulation of inflammation, cell stemness, and insulin secretion [26–28]. *miR-187* was shown in our preliminary screening study as the 3rd most conspicuously up-regulated miRNA in HNSCC [7]. However, the oncogenic role of *miR-187* and its target gene in OSCC have been unknown.

*BARX2* (aka, BarH-like Homeobox 2; homeobox protein BarH-like) is located on chromosome 11q24-25 [29]. Frequent loss of 11q23-25 loci has been reported in ovarian cancers and HNSCC [30, 31]. *BARX2* is known to be involved in cytoskeletal organization, cell adhesion, growth factor signaling, and transcriptional regulation, and it acts as a transcription factor [32]. *BARX2* is also involved in the development of craniofacial structures, salivary glands, hair follicles, and the squamous epithelium of the tongue and esophagus [33–36]. Moreover, *BARX2* is reported to be down-regulated in ovarian cancer and hepatocellular carcinoma, indicating that it normally acts as a tumor suppressor [30, 37, 38]. In this study, we investigated the oncogenic role of *miR-187* by targeting the *BARX2* tumor suppressor in OSCC.

## RESULTS

### Increased *miR-187* expression in OSCC tumors and patient plasma

**Figure 1:**
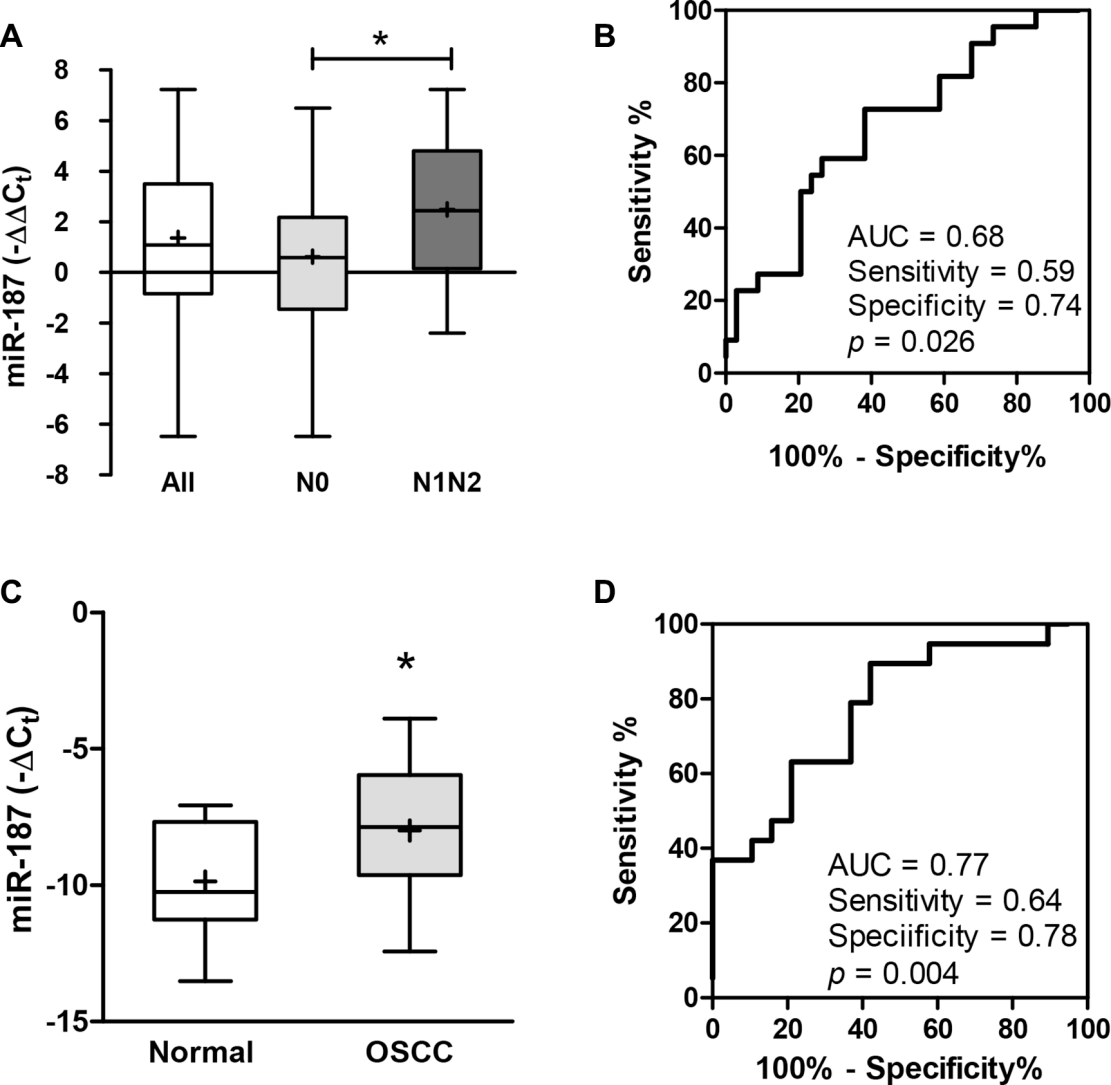
Up-regulation of *miR-187* expression in OSCC (**A**, **C**) Box and whiskers plots illustrating the expression of *miR-187* in tumor tissue pairs (A) and plasma (C) detected by qRT-PCR analysis. Un-paired *t*-test; ^*^p < 0.05. (**B**, **D**) ROC analysis. (B) Comparison across tumors without metastasis and those with metastasis. (D) Comparison of plasma samples from control individuals and OSCC patients. AUC, area under curve.

To explore the expression of *miR-187*, 56 OSCC tumors and their paired NCMTs were subjected to qRT-PCR analysis (Table S1). Up-regulation of *miR-187* was found in 40 (71%) of OSCC tumor tissues. In addition, a significant increase in *miR-187* expression was noted in tumors with nodal metastasis relative to tumors without node involvement (Figure 1A). ROC analyses indicated that *miR-187* expression in OSCC had a predictive power of 0.68 for distinguishing metastatic from non-metastatic states (Figure 1B). *miR-187* expression was not associated with other clinicopathological parameters. To investigate examine the feasibility of using diagnostic value of the plasma level of miR-187, we compared the plasma levels as a diagnostic marker, plasma samples were collected of miR-187 from in OSCC patients and healthy controls. A significant increase in −ΔCt in patients with OSCC relative to controls was noted (Figure 1C). ROC analysis indicated that the plasma *miR-187* level had a predictive power of 0.77 for distinguishing malignant from non-malignant states (Figure 1D).

### *miR-187* expression increased OSCC oncogenicity

**Figure 2:**
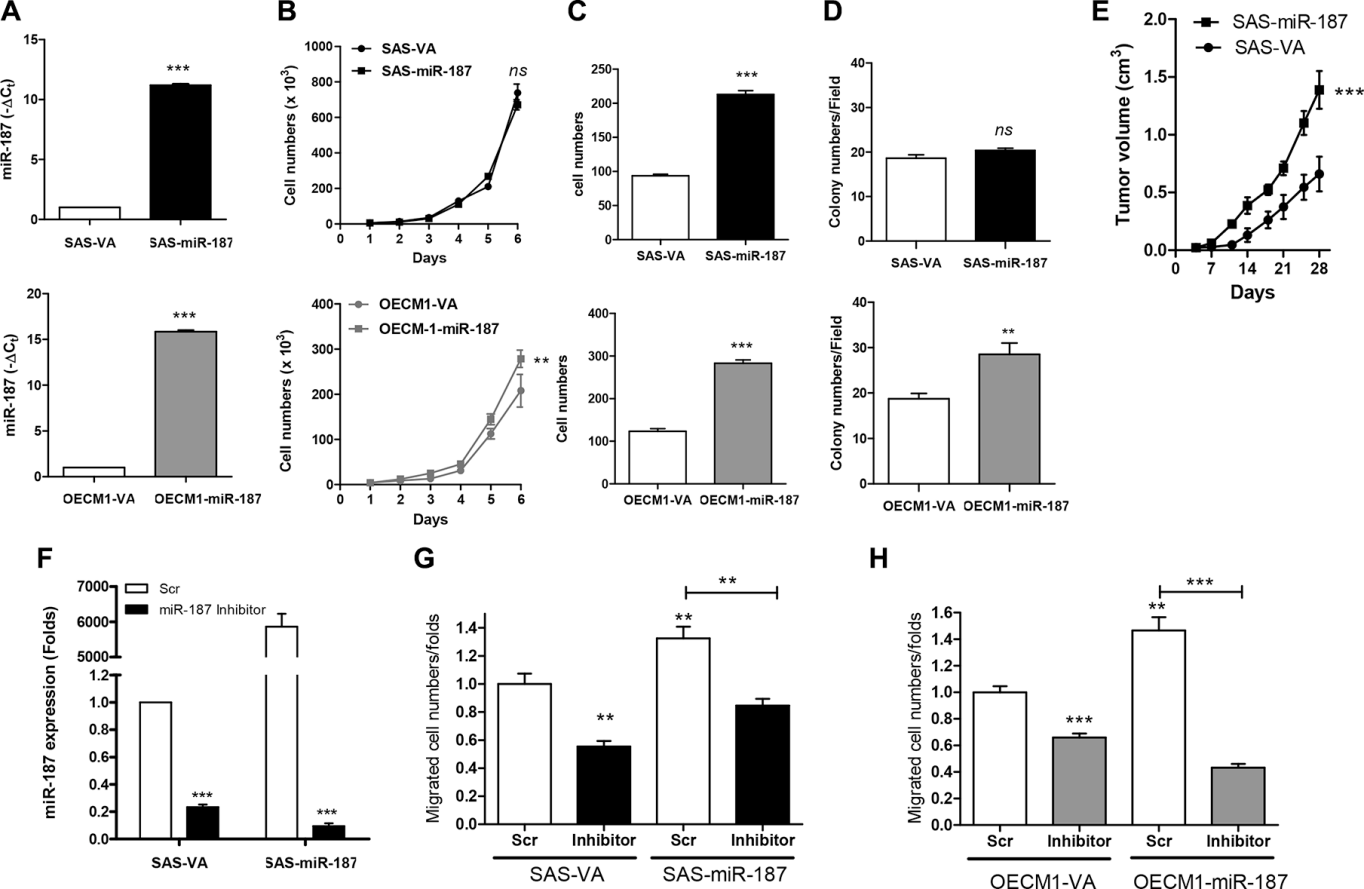
*miR-187* modulates oncogenicity of OSCC cells (**A**) *miR-187* expression. (**B**–**D**) Assays of proliferation, migration and AIG, respectively. (A–D), Upper, SAS cell subclone; Lower, OECM1 cell subclone. (**E**) Subcutaneous tumorigenesis assay for SAS cell subclone. (**F**, **G**) Treatment with *miR-187* inhibitor in SAS cell subclone. (F) *miR-187* expression; (G) Migration assay. (**H**) Migration assay of OECM1 cell subclone following the treatment with *miR-187* inhibitor. Data are presented as the mean ± SE. *ns,* not significant; ^**^p <0.01; ^***^p < 0.001; two-way ANOVA or Mann–Whitney test.

Cell subclones expressing *miR-187* were established in SAS and OECM1 cells, which have high and low endogenous *miR-187* expression, respectively (Figure 2A). These subclones were designated SAS-*miR-187* and OECM1-*miR-187*. The *miR-187* expression in SAS-*miR-187* and OECM1-*miR-187* subclone increased ~11.2 and ~15.9 folds relative to respective control subclones. Exogenous *miR-187* expression did not significantly change the proliferation (Figure 2B, upper) or AIG (Figure 2D, upper) of SAS cells. However, exogenous *miR-187* expression significantly increased the migration (Figure 2C, upper) and xenografic tumor growth (Figure 2E) in SAS-*miR-187* subclones. The proliferation, migration, and AIG in OECM1-*miR-187* subclone was higher than control subclone (Figure 2B–2D, lower). Upon treatment with an *miR-187* inhibitor, endogenous and exogenous *miR-187* expression was drastically suppressed (Figure 2F). The increased cell migration associated with endogenous and exogenous *miR-187* expression was decreased by *miR-187* inhibition in both SAS (Figure 2G) and OECM1 cells (Figure 2H). Overall, *miR-187* expression increased the oncogenicity of OSCC cells, especially in the OECM1 cell line. The phenotype of increased migration was particularly consistent across different OSCC cells.

### Identification of *BARX2* as the target gene of *miR-187* in OSCC

**Figure 3:**
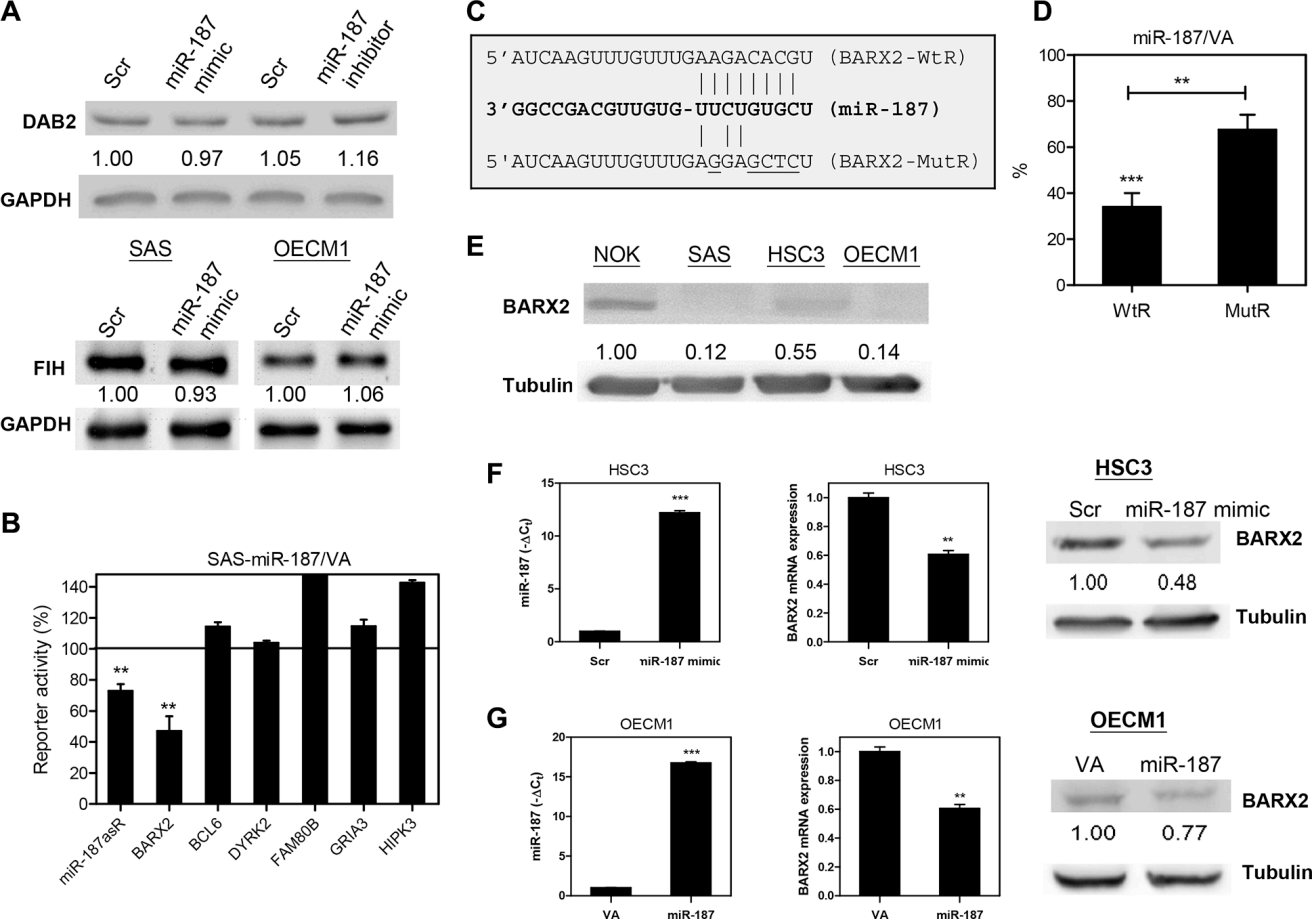
*miR-187* targets *BARX2* in OSCC (**A**, **E**) Western blot analysis. (A) Upper, The treatment with *miR-187* mimic does not affect the Dab2 expression in SAS cells. Lower, *miR-187* mimic treatment does not affect the FIH expression in OSCC cells. (**B**, **D**) Reporter assay. (B) Reporters for various genes. The results indicated that the activity of *BARX2* reporters was repressed by ~55% in SAS-*miR-187* cell subclone relative to control. The *miR-187*asR activity is decreased for ~28%. (**C**) Schematic diagram to show the complimentarity between *miR-187* and *BARX2*-WtR, and *BARX2-*MutR. (D) Suppression of *BARX2-* WtR activity in SAS-*miR-187* cell subclones is partially reversed in *BARX2-*MutR. (E) *BARX2* protein expression in oral keratinocytes. OSCC cell lines exhibited lower *BARX2* expression than did NOK cells. (**F**, **G**) HSC3 and OECM1 cell subclones, respectively. (F) Up-regulation of *miR-187* expression in HSC3 cells (left) following treatment with *miR-187* mimic is associated with decreased *BARX2* mRNA expression (middle) and protein expression (right). (G) Up-regulation of *miR-187* expression in OECM1-*miR-187* cell subclone (left) is associated with decreased *BARX2* mRNA expression (middle) and protein expression (right). Numbers below pictures represent normalized values. Data are presented as the mean ± SE. *ns,* not significant; ^**^p < 0.01; ^***^p < 0.001; Mann–Whitney test.

*Dab2* was reported to be a target of *miR-187* in ovarian cancers [17]. *FIH,* a tumor suppressor gene in HNSCC [7], was predicted to be a target of *miR-187*. However, our preliminary Western blot analyses have excluded these genes as *miR-187* targets in OSCC cells (Figure 3A). To generate reporters for target screening, we cloned sequence fragments of the predicted *miR-187* target genes *BARX2, BCL6*, *DYRK2*, *FAM80B*, *GRIA3,* and *HIPK3*. The assays were validated in side-by-side analysis showing the suppression of *miR-187*asR activity (Figure 3B). Of the potential targets, *BARX2* emerged as a target of *miR-187*, as the reporter activity decreased significantly, to 46% of control reporter activity in SAS-*miR-187* cell subclones (Figure 3B). Figure 3C illustrates the complementarity between the *BARX2* 3′UTR sequence and *miR-187*. We then generated *BARX2* MutR, in which the targeted sites were mutated. Reporter assays of SAS-*miR-187* cell subclone indicated that *miR-187* repressed the reporter activity by directly targeting the wild type sequence and that the mutation partially reverted the repression (Figure 3D). OSCC cell lines had lower *BARX2* expression than did NOK cells (Figure 3E). *BARX2* expression was about 4-fold higher in HSC3 than in SAS and OECM1 cells. To confirm the targeting of *miR-187* on *BARX2*, HSC3 cells were treated with *miR-187* mimic for 48 hr. Exogenous *miR-187* expression (Figure 3F, left) was associated with decreased *BARX2* mRNA expression (Figure 3F, middle) and *BARX2* protein expression (Figure 3F, right) in HSC3 cells. Similarly, the OECM1-*miR-187* cell subclone also had lower *BARX2* expression (Figure 3G).

### Knockdown of *BARX2* expression increased OSCC oncogenicity

**Figure 4:**
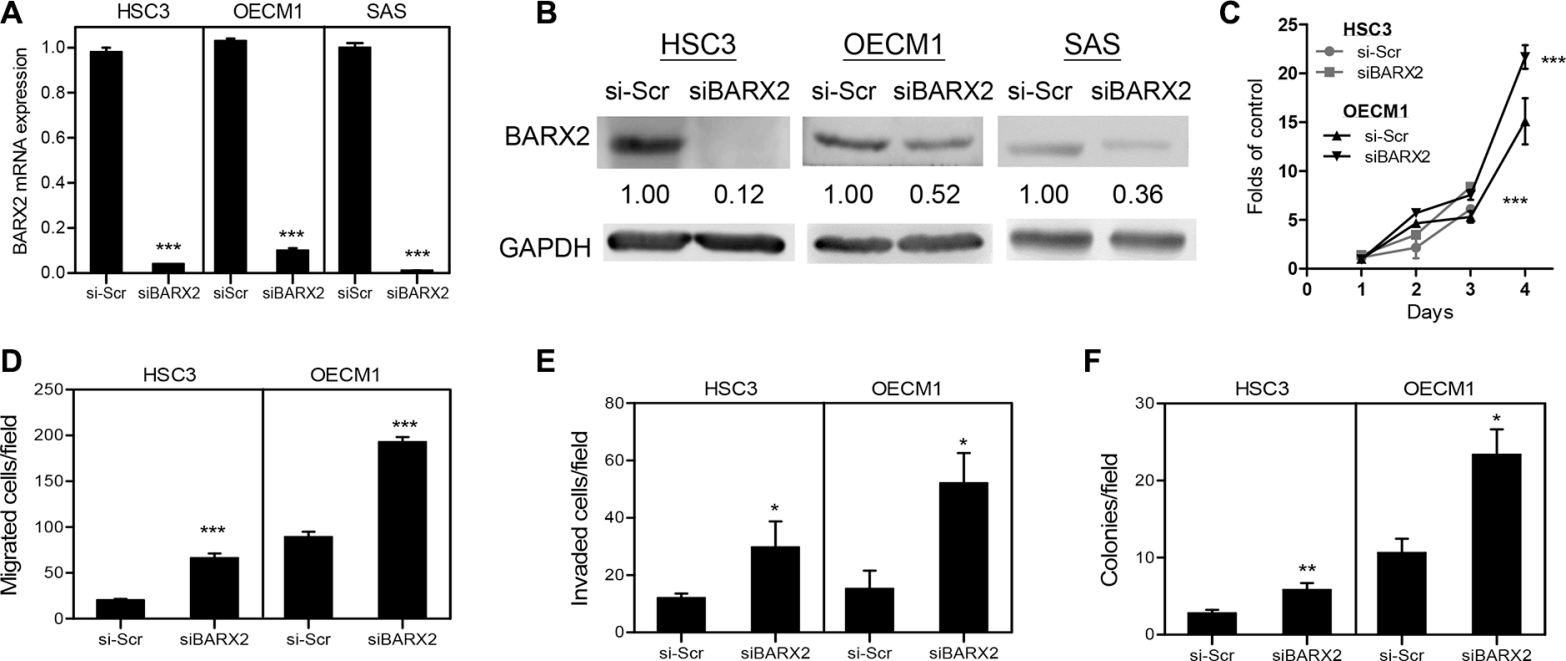
si*BARX2* treatment enhances oncogenicity (**A**, **B**) qRT-PCR analysis and Western blot analysis. Treatment of OSCC cells with si*BARX2* oligonucleotide leads to decreased *BARX2* mRNA expression (A) and protein expression (B). (**C**–**F**) proliferation, migration, invasion, and AIG analysis in HSC3 and OECM1 cells treatment with si*BARX2* oligonucleotide. Numbers below pictures represent normalized values. Data are presented as the mean ± SE. ^*^p < 0.05; ^**^p < 0.01; ^***^p < 0.001; two-way ANOVA or Mann–Whitney test.

OSCC cells were treated with si*BARX2* oligonucleotides for 48 h. qRT-PCR analysis revealed the significant down-regulation of the BARX2 mRNA expression clear down-regulation of *BARX2* mRNA expression (Figure 4A) and protein expression (Figure 4B) in OSCC cells. *BARX2* knockdown was greater in HSC3 cells than in OECM1 cells and was associated with a significant increase in proliferation (Figure 4C), migration (Figure 4D), invasion (Figure 4E) and AIG (Figure 4F) in both HSC3 and OECM1 cells.

### Exogenous *BARX2* expression decreased OSCC oncogenicity

**Figure 5:**
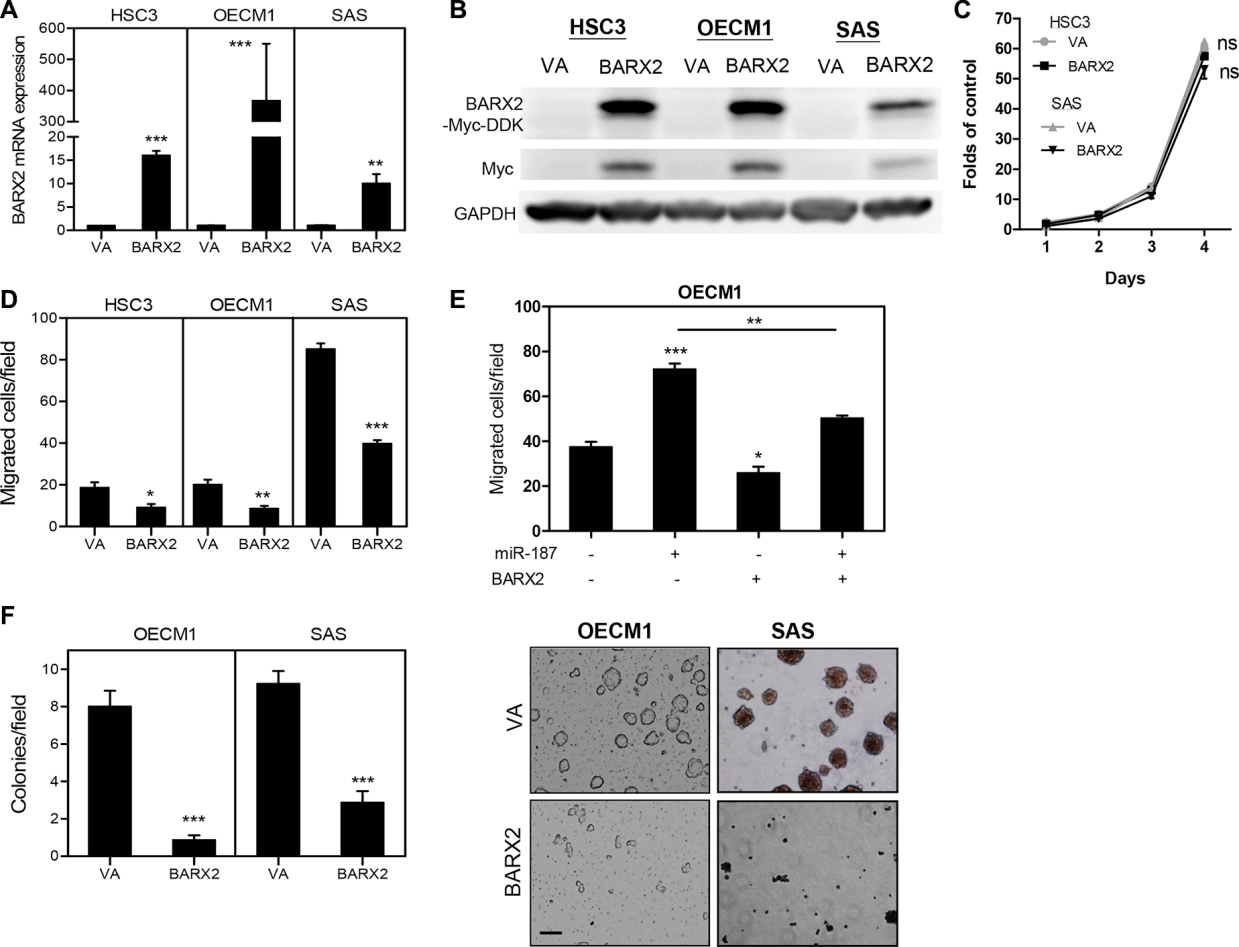
OSCC cells expressing *BARX2* exhibit decreased oncogenicity (**A**, **B**) qRT-PCR analysis and Western blot analysis. *BARX2* mRNA expression (A) and exogenous *BARX2*-myc-DDK protein expression (in B) are increased in OSCC cell subclones with stable exogenous *BARX2* expression. (**C**, **D**) proliferation and migration. (**E**) Migration assay in OECM1 cells. BARX2 expression reverted *miR-187-*induced cell migration. (**F**) AIG assays. Representative fields of colonies (right) and quantitation (left); bar, 50 μm. Data are presented as the mean ± SE. *ns,* not significant;^*^p < 0.05; ^**^p < 0.01; ^***^p < 0.001; two-way ANOVA or Mann–Whitney test.

As *BARX2* knockdown promoted oncogenicity, the effects of *BARX2* in suppressing oncogenicity were further tested in OSCC cell subclones expressing the *BARX2*-myc-DDK fusion protein (Figure 5A, 5B). Exogenous *BARX2* expression had no effect on proliferation (Figure 5C) but decreased the migration of OSCC cells (Figure 5D). OECM1 cells expressing BARX2 were treated with *miR-187* mimic. Data showing that *miR-187-*induced cell migration was attenuated by BARX2 (Figure 5E) support the notion that *miR-187* targeted BARX2 to modulate OSCC cell migration. Exogenous *BARX2* expression also decreased the AIG of OSCC cells (Figure 5F).

### Exogenous *BARX2* decreased orthotopic tumorigenesis of OECM1 cells

**Figure 6:**
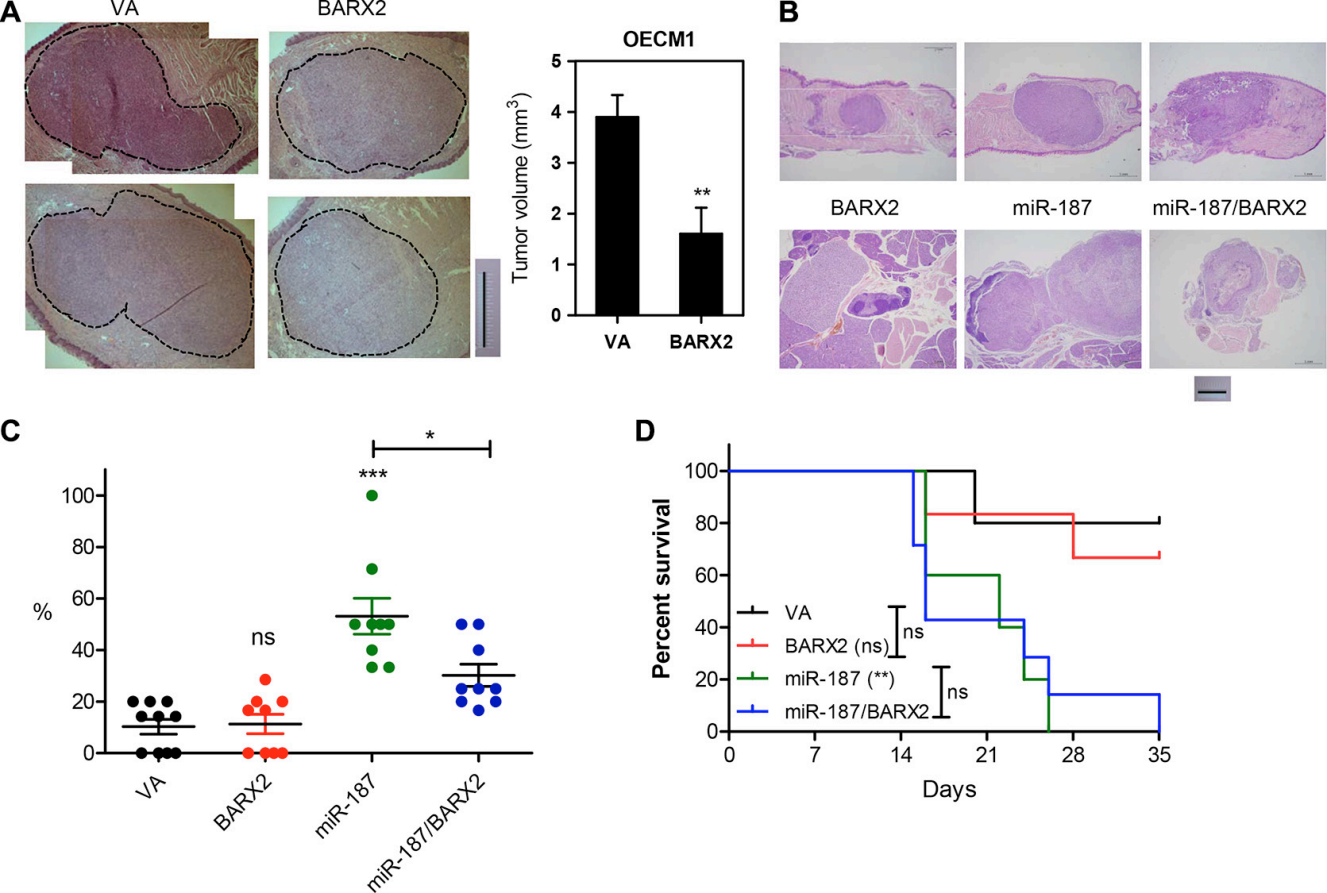
Analysis of tumor xenografts in athymic mice (**A**) Orthotopic tongue xenografts in OECM1 cell subclones. Illustrations of representative tongue sections (left). Dotted lines indicate tumor areas; bar, 1 mm. Quantitation of tumor volume (right). (**B**–**D**) Orthotopic tongue xenografts and neck metastasis in SAS cell subclones. (B) Illustrations of representative sections of primary tumors in tongue and neck nodal lesions. (C) Dot plot of metastatic rate of cell subclones. Mann–Whitney test. (D) Kaplan-Meier survival analysis of cell subclones. Data are presented as the mean ± SE. *ns,* not significant; ^*^p < 0.05; ^**^p < 0.01; ^***^p < 0.001.

Orthotopic tongue tumorigenic assays were carried out on OECM1 cell subclones expressing BARX2 [10]. Exogenous BARX2 expression significantly reduced the growth of orthotopic xenografts (Figure 6A), supporting the hypothesis that *BARX2* is a suppressor of OSCC oncogenicity, particularly in regard to cell mobility. Although there was significant difference in the primary tumor burden, gross evaluation of dissected neck tissue did not reveal potential metastasis in mice. Moreover, the survival rate of OECM1 cell subclones expressing BARX2 is not different from controls.

### Exogenous *BARX2* rescued *miR-187*-enhanced neck metastasis of SAS cells

The tissue analysis revealed the primary xenografic tumor growth of SAS cell subclones in tongue and the metastatic involvement in neck lymph nodes (Figure 6B). Since the advanced tumor may confound the metastasis rate, mice carrying primary tumors > 20 mm^3^ were excluded for metastasis analysis [10]. In animals carrying relatively smaller tumors, *miR-187* expression significantly increased the neck metastasis, and this increase can be rescued by concordant BARX2 expression (Figure 6C). However, exogenous BARX2 expression did not affect the metastatic rates of control cell subclone. The survival rate of mice carrying the xenografts of SAS-*miR-187* cell subclones was drastically decreased comparing to controls (Figure 6D). Exogenous BARX2 expression did not seem to affect the survival in this animal model.

### Decreased *BARX2* expression is associated with the advanced nodal metastasis of OSCC

**Figure 7:**
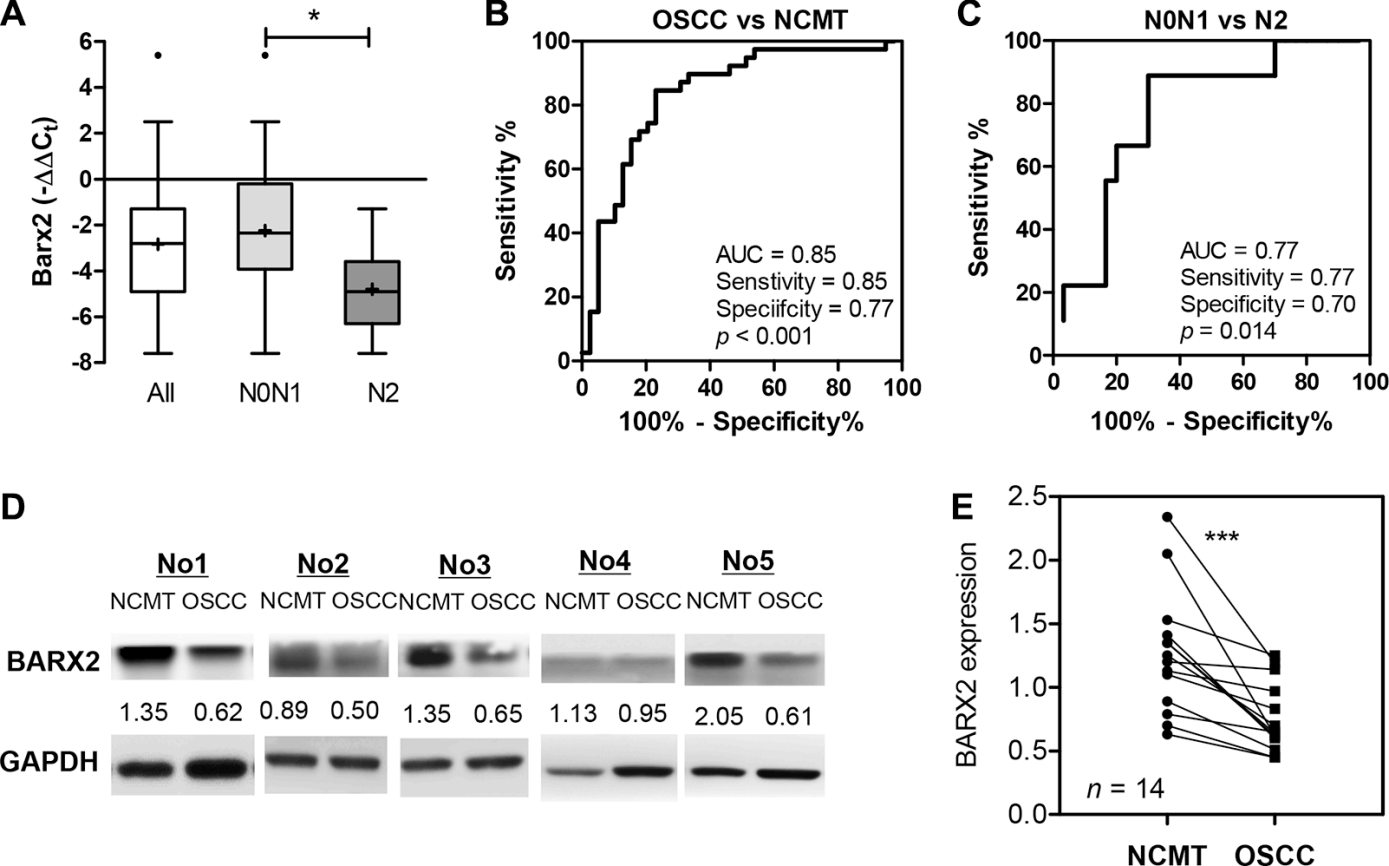
Down-regulation of *BARX2* expression in OSCC (**A**) Box and whiskers plots illustrating the down-regulation of *BARX2* mRNA expression in tumor tissue pairs as detected by qRT-PCR analysis. The down-regulation was particularly evident in tumors with advanced neck metastasis (N2). (**B**, **C**) ROC analysis. (B) Comparison between NCMT and tumors. (C) Comparison across tumors with N2 grade nodal metastasis and others. AUC, area under curve. (**D**, **E**) BARX2 protein expression in OSCC. (D) Western blot analysis of *BARX2* protein expression in 5 representative NCMT and OSCC sample pairs. Numbers below pictures represent normalized values. (E) Quantitation of 14 sample pairs. Paired *t*-test; ^***^p < 0.01.

The expression of *BARX2* in tumors and their paired NCMTs were analyzed using qRT-PCR analysis and Western blot analysis. A reduced expression of *BARX2* mRNA was found in tumor tissues relative to the surrounding tissue. Although there was no significant difference in *BARX2* mRNA expression on metastatic or recurrent tumors, a significant decrease in *miR-187* expression was seen in tumors with advanced nodal metastasis (N2) relative to contrasting tumors (Figure 7A). ROC analysis indicated that *BARX2* mRNA expression in OSCC had a predictive power of 0.85 for distinguishing OSCC from NCMT (Figure 7B), and a power of 0.77 for distinguishing the most severe metastatic status from other states (Figure 7C). Analysis of *BARX2* protein expression in 14 available tissue pairs also revealed significant down-regulation of *BARX2* protein expression in OSCC tumors (Figure 7D, 7E).

## DISCUSSION

*miR-187* has diverse expression across malignancies and plays differential oncogenic roles in different types of tumors [15, 16, 18–25]. Our previous screening study identified *miR-187* up-regulation in HNSCC [7], and the present study further confirms the up-regulation of *miR-187* in OSCC tissues and correlates *miR-187* up-regulation with lymph node metastasis. Functional clues support the oncogenicity of *miR-187* in OSCC, particularly with respect to enhancing tumor cell migration. Exogenous *miR-187* expression levels differ between cell lines, which may underlie the differences in its potential to promote proliferation and AIG. *miR-187* expression also increases the metastatic rate of xenografic tumors and this is associated with worse host survival. The findings in animal models seem to be consistent with the clinical implications.

Many oncogenic miRNAs are potential serological markers of HNSCC or OSCC [2, 10, 39], and our preliminary analysis further supports that plasma *miR-187* is a potential marker of OSCC. Further study is required to confirm the efficacy of *miR-187* as a non-invasive marker of oral premalignant disorders [40]. *miR-21*, *Let-7* family members, *miR-133a* and other miRNAs are involved in the metastasis machinery of HNSCC by targeting regulators of cell stemness or mobility [3–6, 8, 9]. It would be important to specify the enrichment of these aberrant miRNAs with *miR-187* in promoting tumor metastasis in future study.

Few studies have investigated the relationship of *miR-187* targets to tumorigenesis [7, 17, 24]. The targets and mechanisms associated with *miR-187* in OSCC pathogenesis are unknown. Studies have excluded *Dab2*, *FIH* suppressors, and other predicted genes as *miR-187* targets [7, 17]. *BARX2* is involved in regulating squamous epithelium and craniofacial development [33–36] and is a suppressor of several type of neoplasms [30, 37, 38]. This study provides novel evidence that *BARX2* is a target of *miR-187* in OSCC. Loss of heterozygosity in loci containing 11q24.2, which harbors the *BARX2* gene, is frequent in HNSCC [41]. We observed that OSCC cell lines exhibited decreased *BARX2* expression compared to that of NOK cells, possibly due to allele loss or targeting by endogenously-expressed *miR-187* in cancer cells. Furthermore, knockdown and overexpression experiments indicate suppressor activity of BARX2 in multiple OSCC cell lines. Our findings suggest that in addition to chromosomal deletion, epigenetic regulation by oncogenic miRNAs such as *miR-187* is also able to repress BARX2 in OSCC pathogenesis.

With modest up-regulation of *miR-187* in OSCC, qRT-PCR analysis and Western blot unequivocally demonstrated a drastic down-regulation of BARX2 expression in OSCC tumors relative to paired normal mucosa. This decreased expression could be caused by the combined effects of BARX2 deletion and the targeting effects of *miR-187*, since *miR-187* was not greatly up-regulated in cancer cells relative to normal cells. Intriguingly, BARX2 down-regulation was particularly apparent in tumors with advanced nodal metastasis. In agreement with this fact, BARX2 expression reduces the tumor metastasis in xenografic tumors exhibiting highest metastatic potential. This finding suggests that BARX2 expression could be a marker for OSCC metastasis and that restoration of BARX2 could be used as a therapeutic regimen to intercept the progression of OSCC. Acting as a transcription factor, BARX2 is reported to control the expression of adhesion molecules and cytoskeletal elements [32, 36, 37], which may underlie its repressive activity against cell mobility and invasion. Although BARX2 expression reduces the tumor burden of xenografts, it is unable to decrease the mortality of animals significantly, its targets and functional mechanisms in OSCC tumors remain to be identified. Alternatively, the rather low abundance of exogenous BARX2 expression in SAS cells or the relatively smaller sample being analyzed in this study could have impeded the analytical power.

This study suggests that *miR-187* may contribute to OSCC progression through suppression of *BARX2* expression and indicates the clinical importance of the *miR-187*–BARX2 cascade in tumor metastasis. A further understanding of the signals that up-regulate *miR-187* expression and downstream effectors of *BARX2* is needed to clarify the role of this regulatory axis in neoplastic processes. Such information will be valuable for the development of diagnostic tools and therapies.

## MATERIALS AND METHODS

### Tissue and blood samples

The surgical specimens consisted of the primary OSCC tumors together with paired non-cancerous matched tissues (NCMTs) (Table S1). Blood samples (5 mL) were collected from patients one week before surgery and from 19 sex- and age-matched control subjects with no oral disease or malignancy. These samples were collected after obtaining written informed consent, and this study was approved by The Institutional Review Board (IRB) of Mackay Memorial Hospital with IRB approval numbers 09MMHIS146 and 10MMHIS185.

### Cell culture, reagents and phenotypic analysis

The HSC3, OECM1, and SAS OSCC cell lines, 293FT cells, phoenix package cells, and NOK cells established in our laboratory were cultured as previously described [7, 42]. An *miR-187* mimic, *miR-187* inhibitor, and scramble (Scr) control were purchased from Applied Biosystems (Foster City, CA, USA). The si*BARX2* oligonucleotide and scramble (si-Scr) control oligonucleotide were purchased from Santa Cruz Biotechnology (Santa Cruz, CA, USA). TransFectin Lipid Reagent (BioRad Laboratories, Hercules, CA, USA) was used as the transfection reagent. Analyses of oncogenic phenotypes, including cell proliferation, transwell migration assay, cell invasion and anchorage-independent growth (AIG) were carried out according to previously published protocols [7, 10, 42].

### Establishment of cell subclones with *miR-187* or *BARX2* expression

A lentivirus carrying the *pre-miR-187* sequence and a red fluorescence (RFP) tag was purchased from Biosetta (San Diego, CA, USA). Red fluorescence in cells indicated infection. OSCC cell subclones with stable *miR-187* expression and controls were established by puromycin selection. The coding sequence of *BARX2* together with Myc-DDK tag were cloned into pBabe-puro retroviral vector to produce the pBabe-*BARX2*-myc-DDK construct. Cell subclones with *BARX2* expression were established by viral infection and puromycin selection. In addition, cell subclones with exogenous *miR-187* and BARX2 expression (designated *miR-187*/BARX2) were established after viral infection, puromycin selection and fluorescence sorting.

### qRT-PCR analysis

Tri-reagent was used to isolate total RNA, which was reverse transcribed to produce the corresponding cDNAs. The TaqMan miRNA assay kit was used to quantify the expression of *miR-187, BARX2,* and other genes according to the manufacturer's instructions (Applied Biosystems). *Let7a* small nuclear RNA, *U6B,* or *GAPDH* were used as an internal controls

### Western blot analysis

Cell lysates (60 μg) were subjected to Western blot analysis using various primary antibodies (Table S2) and secondary antibodies (Chemicon Int., Billerica, MA) according to previously described protocols [7, 42]. Normalization of the signals to internal GAPDH or tubulin was used to generate relative expression values.

### Prediction of targets

Targetscan *in silico* (http://www.targetscan.org/) version 6.2 was used to predict the potential targets of *miR-187* [7].

### Reporter plasmid construction and assays

To test the targeting activity of *miR-187*, fragments of 3′ UTR sequences of *BARX2*, *BCL6*, *DYRK2*, *FAM80B*, *GRIA3,* and *HIPK3* containing predicted *miR-187* target sites were amplified by PCR (Table S3) and cloned into the pCMV-LacZ plasmid [7]. In addition, the antisense sequence of *miR-187* was cloned to generate *miR-187*asR as a positive control reporter. The wild type 3′UTR sequence of *BARX2* was also cloned into the pMIR-REPORT vector (Life Technologies, Grand Island, NY, USA) (Table S4) to generate the *BARX2* WtR reporter [43]. A mutant reporter (MutR) construct was obtained from the *BARX2* WtR reporter by replacing the original sequence AGACACG at the target site of *BARX2* 3′UTR with GGAGCUC to create a new Sac I restriction enzyme digestion site. LacZ activity, firefly luciferase activity, or normalization to transfection efficiency was used to represent reporter activity.

### Tumorigenesis and metastasis assays

For xenografic tumorigenesis, 2.5 × 10^5^ SAS cell subclones were injected subcutaneously into the flank of 6–8-week-old athymic mice for 4 weeks. For orthotopic induction of xenografts in tongue, 3 × 10^5^ OECM1 cell subclones were injected into the central portion of tongue in mice for 2 weeks. After sacrifice of the animals, the resected tumors, tongues and neck tissues were photographed, and examined grossly and histopathologically. Tumor volumes were calculated using the formula: volume = *0.5* × *a* × *b*^2^, where *a* and *b* were the long and short diameters of the tumors, respectively [7].

For induction of neck nodal metastasis, 3 × 10^5^ SAS cell subclones were injected into the central portion of the tongue of 6–8-week-old athymic mice. The mice were sacrificed at the 5th week after inoculation or at the time points when body weight loss for more than 30% [10]. Indian ink was injected into the tongue tissue to facilitate node identification. In addition, the head and neck region was photographed under an illuminating device (LT9500 Illumatool TLS; Lightools Research, Encinitas, CA) to detect positive nodes. The whole tongues were resected and subjected to histopathological evaluation. Neck tissues achieved by radical dissection were embedded for histopathological examination. The animal study was approved by the Institutional Animal Care and Use Committee of Mackay Memorial Hospital.

### Statistical analysis

The *t-*test, Mann–Whitney test, and two-way ANOVA were used to compare differences between variants. The extent to which variants could be used to distinguish disease status was determined using receiver operating characteristic (ROC) analysis. The area under the curve (AUC) showed the discriminative ability. Kaplan-Meier analysis was used for the comparison of survival between groups. A difference was considered statistically significant when *p* < 0.05.

## SUPPLEMENTARY MATERIALS FIGURES


