# Supplementary material for: Up-regulation of miR-187 modulates the advances of oral carcinoma by targeting BARX2 tumor suppressor
Source: Oncotarget. 2016 Aug 17;7(38):61355–65. doi: 10.18632/oncotarget.11349 (PMC5308656; doi:10.18632/oncotarget.11349)
Supplement: Supplementary file 1 [file oncotarget-07-61355-s001.pdf]

Up-regulation of *miR-187* modulates the advances of oral carcinoma by targeting *BARX2* tumor suppressor

Supplementary Materials

Supplementary Table S1: Clinicopathological parameters of the OSCC

n =	**56**
T1, 2	19
T3, 4	37
N0	34
N1	13
N2	9
Stage I, II	15
Stage III, IV	41

Supplementary Table S2: Primary antibodies

Antibodies	Molecular Weight (kDa)	Dilution	Company & Cat #	
BARX2 (S-13)	43	1:200	Santa Cruz Biotech	sc-68144
cMyc (9E10)	67	1:1000	Santa Cruz Biotech	sc-40
Dab2	85	1: 200	BD Biosciences	610465
FIH	40	1: 1000	Santa Cruz Biotech	Sc-26219
GAPDH	36	1:2000	Santa Cruz;	sc-32233
Tubulin	**50**	**1: 5000**	Sigma-Aldrich	T51681

Supplementary Table S3: The primers used to generate reporter constructs by PCR amplification

BCL6 reverse: CCGAAGCTTTTCAAGTCCCTGTGTCTG
DYRK2 forward: CCGTCTAGAGATCTGGTCGGTAAAGG
DYRK2 reverse: CCGAAGCTTCCACATTCAACCATTTTCAC
FAM80B forward: CCGTCTAGAGGCATTCATCAAGCACATTATC
FAM80B reverse: CCGAAGCTTCTGTCTGAGCACCTACATTTTC
GRIA3 forward: CCGTCTAGAAGAGGAAATCACCGAAAACG
GRIA3 reverse: CCGAAGCTTTGATGGTTTGCATTGTCGC
HIPK3 forward: CCGTCTAGAATGGGAGCAGAAGTCCAG
HIPK3 reverse: CCGAAGCTTGGAACCGGTTTACAATGCAG

Forward and reverse; direction.

Supplementary Table S4: BARX2 3′UTR sequence and reporter construct

Forward primer : 5′ GCGACTAGTCCAGTCATCAAAGCAGAGAG 3′

Reverse primer : 5′ GCGACGCGTGACCTTGAGCAGTAGGATAG 3′

>ENST00000281437 utr3:KNOWN_protein_coding
AGTAAAACCCTTTTGAGGGAAGAGGGAGACTGGGGAGAAGGGAAAA
GAGAGAAGGCAGGGAGAGTAGGGAGAGAAAACCTTCCAGCAGCCCA
GTAAACTGCGGGCGAAGAGATCTACCCGTCTCCCTCCCTCCCACAGTT
ACCATTGGCCTTGTCATCGCAAGCATTTGACAAAGACTTGCTTGTCTTG
GGCCTGTCACCTCCTGAAAGGCTGCTTTAGCTGTGGATGCCCTTGATTA
AGGGAGAGAGCGCCTAGGAGCTGCCTGCCCCAGCTGGGGTGACGGCTG
TAGGGCTGGGTCTATGTTGCAAGCCCTATATCCTAGCATGCAGTGGAAA
GTGCTTAGCTCTCTCCCTCCTGACCTCTGGGCAGCCAGTCATCAAAGCA
GAGAGACGTGGCGGCATGTGGGCAGCATGCCCAGGTTCCTTGCTGACTC
AGCACTTATTTCTGTAGTTTTAAAAAAGAATTTAATGTTTTTGGTTGTATT
TTTTTGGGGGGGTGAGGGTGGGCAAAAACATGGGGGTAGTTCTGAGTTG
TTAGAAATGTTTCTGAATCAAGTTTGTTTGAAGACACGTGTGCCTTTGTA
CCCATTATAAGATGGTCATAAGACCCAAGAACTGATAAGCTTTGGTTTTT
TTTTTGTTTTGTTTTGTTTTTTGCTTCATTTACCCATTCATGCCTAGGGTTC
CATTATTGGAACCCTAAGCTTGTGGGAGTTATTTCTATCCTACTGCTCAA
GGTCATCACCAAGATCTGATTTTTCATAAAAAAACATTTGTGACCTTCGGC
ATAAATGGGTTAAGGTGCCATCCCTGAAACTGCAATGCAGATATGTTCA
GATAACTTTTATTTTTTAATTAAAAATAAATCTTTCAAAA

**miR-187* binding site underlined.
